# Standard requirements for GCP-compliant data management in multinational clinical trials

**DOI:** 10.1186/1745-6215-12-85

**Published:** 2011-03-22

**Authors:** Christian Ohmann, Wolfgang Kuchinke, Steve Canham, Jens Lauritsen, Nader Salas, Carmen Schade-Brittinger, Michael Wittenberg, Gladys McPherson, John McCourt, Francois Gueyffier, Andrea Lorimer, Ferràn Torres

**Affiliations:** 1 Coordination Centre for Clinical Trials, Heinrich-Heine University Duesseldorf, Duesseldorf, Germany; 2 Clinical Trials and Statistics Unit, Institute of Cancer Research, Sutton, London, UK; 3 Dept. of Biostatistics, Odense University, Odense, Denmark; 4 Copenhagen Trial Unit, Rigshospitalet, Copenhagen, Denmark; 5 Coordination Centre for Clinical Trials, Philipps Universität, Marburg, Germany; 6 Health Services Research Unit, University of Aberdeen, Aberdeen, UK; 7 Dublin Centre for Clinical Research, Dublin, Ireland; 8 Centre d'Investigation Clinique, Hôpital Cardiologique Louis Pradel, Lyon, France; 9 Centro Studi A.N.M.C.O, Firenze, Italy; 10 Hospital Clinic I Provincial de Barcelona, Barcelona, Spain

## Abstract

**Background:**

A recent survey has shown that data management in clinical trials performed by academic trial units still faces many difficulties (e.g. heterogeneity of software products, deficits in quality management, limited human and financial resources and the complexity of running a local computer centre). Unfortunately, no specific, practical and open standard for both GCP-compliant data management and the underlying IT-infrastructure is available to improve the situation. For that reason the "Working Group on Data Centres" of the European Clinical Research Infrastructures Network (ECRIN) has developed a standard specifying the requirements for high quality GCP-compliant data management in multinational clinical trials.

**Methods:**

International, European and national regulations and guidelines relevant to GCP, data security and IT infrastructures, as well as ECRIN documents produced previously, were evaluated to provide a starting point for the development of standard requirements. The requirements were produced by expert consensus of the ECRIN Working group on Data Centres, using a structured and standardised process. The requirements were divided into two main parts: an IT part covering standards for the underlying IT infrastructure and computer systems in general, and a Data Management (DM) part covering requirements for data management applications in clinical trials.

**Results:**

The standard developed includes 115 IT requirements, split into 15 separate sections, 107 DM requirements (in 12 sections) and 13 other requirements (2 sections). Sections IT01 to IT05 deal with the basic IT infrastructure while IT06 and IT07 cover validation and local software development. IT08 to IT015 concern the aspects of IT systems that directly support clinical trial management. Sections DM01 to DM03 cover the implementation of a specific clinical data management application, i.e. for a specific trial, whilst DM04 to DM12 address the data management of trials across the unit. Section IN01 is dedicated to international aspects and ST01 to the competence of a trials unit's staff.

**Conclusions:**

The standard is intended to provide an open and widely used set of requirements for GCP-compliant data management, particularly in academic trial units. It is the intention that ECRIN will use these requirements as the basis for the certification of ECRIN data centres.

## Background

Clinical Data Management Systems (CDMS) are used more and more to handle the increasing amount of data that must be collected, processed and analysed in clinical research, whether that data is initially captured remotely and directly from clinical sites using Remote Data Capture (RDC), or using more traditional paper based methods [1]. The development and maintenance of an appropriate data management environment is a tough challenge for academic clinical trials units. A recent survey showed several problems with data management systems in clinical trials conducted at academic centres [2]:

a) There is considerable heterogeneity in the use of different software products for data management, and often proprietary solutions are in place rather than open source or industry supported commercial products.

b) There are deficits in the quality of data management, including in computer system validation.

c) Most centres are constrained by limited human and financial resources in providing adequate levels of data management.

d) The complexities of running a local IT/data management centre, especially for international clinical trials, are underestimated.

e) There exists no widely recognised, specific, practicable and open standard for GCP-compliant data management and the accompanying IT infrastructure.

To expand upon the last point: GCP requirements on data management are mostly unspecific at the technical level [3]. EU Directive 2001/20/EC [4], EU Directive 2005/28/EC [5] and Annex 11 [6] define GCP compliance for clinical trials but specify only a few technical requirements for data management (e.g. necessity for data privacy, security system, system descriptions). The FDA Guidance for Computerized System Used in Clinical Trials [7] or 21 CFR Part 11 [8] covering electronic records and electronic signatures are legally binding in the US but have less relevance for the EU. Similarly, specific regulations exist in many EU countries and several national guidance documents for IT are available (e.g. UK, Germany, Denmark) but with limited or no relevance for other countries [9-12].

For computer system validation purposes a number of additional guidelines are in use for specific aspects of data management, like the PIC/S Guide [13], which defines requirements from the inspectors' point of view and the GAMP^® ^guide [14] defining best practices for system validation. On the other hand, ISO standards cover only the general level of IT infrastructure aspects (e.g. ISO 27001, security management system [15]).

The Good Clinical Data Management Practices (e.g. version 4) [16], proposed as an industry standard for clinical data management, consists of best business practice and acceptable regulatory standards but is mostly intended for GCP compliance training. Members of the Society of Clinical Data Management (SCDM) can download the guide, non-members may purchase the copyright protected document.

In summary, there is no standard for GCP-compliant data management and the underlying IT infrastructure available, which is both generally applicable and practical, as well as being open and available free of charge.

The European Clinical Research Infrastructures Network (ECRIN) [17] is an EU-funded ESFRI- (European Strategy Forum on Research Infrastructures) roadmap project in biological and medical sciences, designed to support clinical research in Europe through providing consulting and other services to investigators and sponsors for multinational clinical trials. ECRIN provides a not-for-profit platform for the support of pan-European clinical research projects. It does this by connecting national networks of clinical research centres (CRCs) and clinical trial units (CTUs), working across all disease areas.

To support clinical trials ECRIN intends to provide a combined DM- and IT-framework using data management systems located in dedicated ECRIN data centres. As part of realising this aim, ECRIN recognised the need for a set of clear standards for data management and the associated IT infrastructure - to guarantee GCP-compliant, efficient, high quality and secure operations in those data centres. Within ECRIN a certification procedure will be generated for data centres based upon the standards presented in this publication.

## Methods

### Overview

Standard requirements were formulated by expert consensus of the ECRIN Working Group on Data Centres. The consensus was developed by a structured and standardized process, coordinated by the chairman of the group and two subgroup leaders. The approach consisted of the following features:

a) a large panel of 25 DM experts with a variety of national origins (11 countries), high expertise and professional background (see list of Working Group members).

b) open discussion by experts (written and orally) with feedback of all responses to all experts.

c) iterations with several rounds of consensus building based on strict versioning of documents with tracking of changes.

d) highly structured representation of requirements as a standardised list of statements.

In the section "discussion" this methodological approach is compared to a more formal consensus development method (e.g. Delphi method, nominal group technique).

### Considered regulations and guidelines

**Figure 1:**
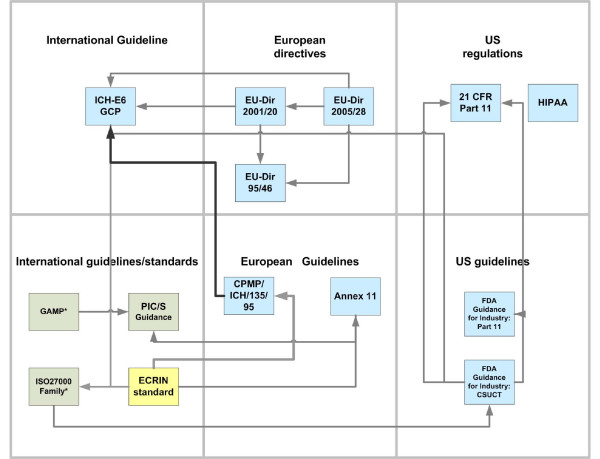
**Standards and guidelines related to GCP**. International and European regulations and guidelines that are relevant for GCP-compliant computer systems and data management are displayed as small boxes. Arrows connecting these documents indicate important references from one document to another (e.g. the arrow from EU-Dir 2005/28 to EU-Dir 95/46 means that EU-Dir 2005/28 refers to EU-Dir 95/46). The correspondence of ICH E6 GCP and European CPMP ICH/135/95 is indicated by a bold arrow.

In developing the ECRIN standard, compliance with ICH GCP [3] was seen as the central requirement. The European directives and guidelines dealing with clinical research all refer to this document (Figure 1), indicating the central importance of GCP for data management in clinical trials. ICH GCP section 5 [3] describes some requirements for the use of electronic trial data and computer systems, e.g. the sponsors operating such computer systems must validate their systems, maintain SOPs for their use, ensure an audit trail for each data change and provide for data security. In addition, further relevant documents were consulted. In summary, the following documents were taken into consideration.

#### International

- ISO 27001 Information Security Management - Specification [15]

#### European

- EU Directive for the implementation of GCP 2001/20/EC [4],

- EU Directive 2005/28/EC [5],

- EU Directive 95/46/EC [18],

- Computerized Systems, EudraLex - Volume 4, Annex 11 [6],

- EMEA Reflection paper on expectations for electronic source documents used in clinical trials [19],

- ECRIN deliverable D10: GCP-compliant data management in multinational trials [20],

#### Other international documents

- Good practice for computerised systems in regulated GXP environments, PIC/S Inspectors Guide [13],

- Good Clinical Data Management Practice, Version 4 of the Society for Clinical Data Management, October 2005 [16],

- Good Automated Manufacturing Practice (GAMP^R^) Version 5 of the International Society for Pharmaceutical Egineering (ISPE) [14].

#### National

- Implementation of Good Clinical Practice Software (University of Southern Denmark) [9],

- German Coordinating Centres for Clinical Trials Networks Policy Document [10],

- Data and Information Management Systems Project (DIMS) - System Standards of UKCRC/NIHR (UK) [12],

- IT-Grundschutz Methodology of the Bundesamt für Sicherheit in der Informationstechnik (BSI) [11], and

- FDA: Guidance for Industry: Computerized Systems Used in Clinical Trials [7] and 21 CFR Part 11 [8].

These documents stimulated discussion and ideas in the Working Group, and sometimes directly suggested a requirement statement. Thus, for example, the requirements of Annex 11 and 21 CFR Part 11 for a clinical data audit trail and the insistence of ISO 27001 that a security management system must be in place could both be included in the requirements list. We found, however, that statements were often too general when it came to the need of specific clinical trial processes and structures. In general, therefore, we have concentrated on defining standard requirements that, whilst certainly in line with the documents listed above, and with the principles of GCP in particular, reflect the special needs of data centres in clinical trials units.

Differences in national regulations of data management for clinical trials were also discussed in the Working Group; for example, specific regulatory requirements and standards are necessary for IT support of clinical trials in Denmark, UK, Germany, and other countries. In Denmark, for example, special standards have had to be applied for the implementation of the infrastructure at the Copenhagen trial centre, which in turn has made the application of certain ISO-standards necessary. To avoid the generation of an overly complex document, the Working Group decided to aim for an Europe wide document, not considering specific national standards but phrasing the requirements in such terms that most national specifications could be covered. Thus the requirements were kept as generic as possible but also specific enough to be useful for the conduct of a data centre to support clinical research.

### Process of expert consensus

Based on requirements extracted from the documents listed above, an already existing ECRIN document on GCP-compliant data management in multinational trials [20], and especially with help from the groundwork of the UK DIMS Project Team [12], a requirements catalogue for GCP-compliant computer system based data management was developed based on expert consensus and formulated as a standard.

To structure the consensus process, the ECRIN Working Group was divided in two subgroups: an Information Technology (IT) subgroup and an Data Management (DM) subgroup, each group working on different requirements. This divison was useful in distinguishing general IT aspects from trial related aspects of data management, though interactions between these two areas were also taken into consideration.

A computer system used for clinical trials cannot be regarded as an isolated application. It must be seen as part of the IT infrastructure of the trial centre using it. The IT subgroup therefore looked at various aspects of that infrastructure as well as systems specifically designed to support clinical trials. Similarly, the DM group examined not just trial specific components of data management systems (in particular the clinical data management application) but also the general aspects of data management systems that are used by all trials.

To reach expert consensus, the following procedure was followed strictly in the process of development of the requirements:

- provision of requirements as a list of statements, each statement with an ID number and a precise description, and categorised as either a 'minimal' requirement or 'best practice'

- provision of a draft version of the requirements to the WP-members

- collection of written feedback

- telephone conference or face-to-face meeting with point by point discussion of individual requirements

- provision of an updated version with a track of the changes to all WP members

This cycle was repeated with several iterations (7 telephone conferences). In addition, the group conducted three face-to-face-meetings, one with participation of international accreditation experts (14 September 2009) and a final full day meeting (March 2010) examining all the remaining contentious issues in detail, and in particular the relationships between the IT and DM standards, as well as the final format of the document.

## Results

**Figure 2:**
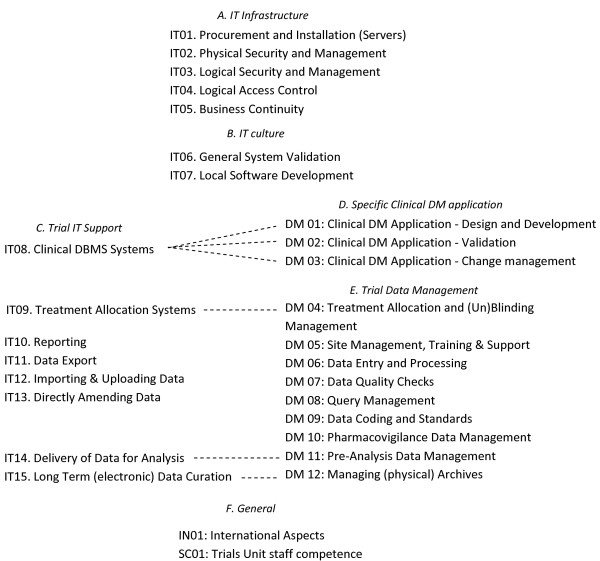
**Relationships between the IT and the DM sections in the ECRIN standard**. The different sections in the IT part and the DM part of the ECRIN standard. Relationships are indicated by arrows (CDMS = clinical data management system, IT = information technology, DM = data management, DBMS = data base management system)

The requirements are divided into an IT and a DM part. In summary, the ECRIN standard covers 115 IT requirements, 107 DM requirements and 13 other requirements. Within each part, there are sections dealing with specific topics, such as "Logical Security and Management (IT03)" or "Query Management (DM08)". Sections are marked by "IT" or "DM" and a number (e.g. DM03). In summary, there are 15 sections related to IT, 12 to DM and two dealing with other aspects. The sections are presented in Figure 2. The first group of sections (IT01 through IT05) deals with the basic IT infrastructure of a data centre, while IT06 and IT07 cover two critical aspects of local IT culture (validation and local software development). The next groups of requirements are more focused on functions specific to data centres; functions generally used (and assessed) within the context of specific trials. Thus, IT08 to IT15 are concerned with IT systems that directly support clinical trial data management. DM01 to DM03 cover the implementation of a specific clinical data management application. DM04 to DM12 address aspects of data management across trials, for example data entry, quality checks and coding.

For each section the requirements are organised as a list of individual requirements with three columns (identification number (ID), e.g. IT03.07), category (minimal or best practice) and textual description). The full set of requirements is available as an attachment to this document (additional file 1).

Though the standard is organised as sections with lists of requirements, the relationships that exist between the sections of the IT and DM parts are rather complex. The relationships are presented in Figure 2. In some cases, obvious links between the IT and the DM sections exists (Figure 2). IT09-DM04, IT14-DM11 and IT15-DM12 are all corresponding pairs of sections, actually dealing with the same topic in each case but from an IT- and DM-perspective respectively. They should therefore be considered together when examining the list. Similarly, IT08 includes some IT specific standards to be expected in clinical database systems, to complement the data management standards of DM01, 02 and 03.

Finally, there are two sections (IN01 and ST01) that deal with more general aspects of the data centre's work and which are not directly connected with the other subgroups. Some of these additional requirements are focused on international aspects (e.g. user support, translation of eCRFs) and others on staff competence (e.g. training, support) but both are necessary to cover support of European wide clinical trials.

Because the standard is set out as sections with lists of requirements with only short descriptions, it can be used easily as a checklist, e.g. for self assessment.

## Discussion

### Standards and requirements for GCP-compliant data management

In establishing a consensus for GCP-compliant data management, the underlying IT infrastructure and the relevant indicators of good GCP practice have been the driving force for the ECRIN Working Group on Data Centres. The development of a standard was initiated by a perceived lack of clarity in this area and the existing heterogenity within academic clinical trial data centres [2]. The aim of this approach and the standard requirements developed is to bring ECRIN and other data centres to the same level of quality and standardisation and to make them evolve towards a common quality level. But there is no claim that the ECRIN standard represents the definitive standard for all clinical trial centres. The validity and practicality of the ECRIN standard will need evaluation by clinical trial units data centres themselves. The scope of the standard is limited to the structure, function and use of computer systems for clinical trials and the value, accuracy and integrity of the data generated and does not cover other aspects of clinical trials. Therefore, the ECRIN standard should not be perceived or managed in isolation from other quality management systems in clinical trial units.

There is no attempt to justify each requirement in any detail, though some are commented more specifically. This paper does not consider the possible consequences of non-compliance in any detail and it does not specify any risk of non-compliance. That is seen as an issue for those who might audit a data management centre.

Clinical data management centres vary considerably in their size, in the available resources and in the extent of quality management [2]; and so some centres are much more likely to have reached certain levels of GCP compliance than others. One important discussion point in the Working Group was that the standard has to reflect what the majority of ECRIN partners or other trial units providing data management services can realistically achieve with their available resources. This is a point of concern for many academic research centres.

For instance, system validation plays an important part in ensuring GCP-compliance of a computer system but can be problematic. Academic units do not, in general, have the resources available in the pharma industry to conduct or outsource a 'full' validation for every system component, including the vendor audit, and to maintain complete change management. In addition, there is no simple way to know how much system validation is necessary or sufficient [21] and the extent and depth of validation required may depend on the interpretation of a particular auditor and whether a commercial software or an inhouse developed software is used. We do acknowledge, however, that system validation probably needs to be improved in many ECRIN centres [2].

Accordingly, the ECRIN standard does not stipulate the best possible IT infrastructure, but instead tries to define minimum requirements and best practice. In this way it allows flexibility wherein centres can develop their resources and improve their operations. Best practices are often worthy of implementation, and may over time become more attainable for all centres. In this way, standards, if they are sufficiently supported and explained, may not only become generally accepted but can also be used as a tool for improvement. At this time it would be impractical, for instance, to insist on the use of specific data standards (e.g. the use of CDISC ODM for metadata) despite their many advantages, especially for international trials [22]. Nonetheless, an updated standard may in future include addtional requirements for metadata export using ODM [23] and centres would be expected to comply with it.

Academic clinical trial units are often part of their corresponding university IT infrastructure. In some cases a part or all of the functionality covered by the standard will not be the direct responsibility of the centre or the trials unit itself, falling instead within the remit of the computer centre of the university or even an outside service provider. For example, IT infrastructure services may be provided by the parent organisation, or a commercial host, or another collaborating trials unit. In such cases, the data centre using the ECRIN requirements should have formal written agreements, e.g. in the form of contracts or service level agreements (SLAs), that ensure that the relevant requirements of the standard will be met by the organisation(s) that provides them.

This attempt to introduce a new standard into the field of computer systems in clinical trials must deal with two related questions: a) What is the justification for a new requirements standard for trials units? and b) how does this new standard fit in with existing requirements for IT systems, validation, data management etc?

The prime justification for the standard stems from the recognition that a deficit in clinical research should result in the establishment of a tool to overcome and improve the situation. Clinical research is dependent upon high quality, reliable and GCP-compliant tools, including appropriate IT and data management systems. However and as decribed in "background", no freely available and sufficiently detailed standard currently exists to evaluate such systems.

Similar difficulties have arisen with several other recent initiatives, e.g. the evaluation of breast cancer centres (EUSOMA) [24] and phase I trial centres (MHRA) [25], or the accreditation of centres for first-in-man studies (JACIE) [26]. In these cases too, requirements were first developed to have a standard available to judge the quality of a structure, process or service. The IT/DM requirements developed by ECRIN follow the same path and offer a quality standard for all interested academic clinical centres that conduct electronic data management in clinical trials to support quality assessment within these centres. This standard is, however, not restricted to academic centres; it may also be useful for commercial software and solutions in the pharmaceutical industry.

### Methodological approach

The way in which this standard fits with other existing requirement schemes has been partly covered in 'Methods' - i.e. it was certainly informed by those other standards, particularly ICH GCP, but has a different focus and level of detail. Existing validation schemes and security audits, for instance, often have an industry focus (and assume industry-level resources). Detailed examination of data management may be based on FDA demands, that again tend to originate in industry and have corresponding expectations. With the ECRIN requirements list, however, we publish a set of concrete requirements for *academic *clinical research centres, within the specific perspective of European clinical trials. In this way the new standard complements the different existing approaches and systems. It provides an easy to use and systematic tool for clinical trial units developing or improving their data management system. Our standard is designed to support the validation efforts of centres by defining agreed, minimal requirements that are GCP compliant and that are realistic for academic units.

In order to develop standards different methodological approaches are used. Formal consensus methods, such as Delphi method or nominal group technique offer a structured, transparent and replicable way of synthesizing individual judgements and have been used, for example, in the development of many guidelines. We did not follow strictly a formal consensus development technique but used certain features of these consensus methods in our structured and standardised consensus process. These features covered iterations and controlled feedback with several rounds based on strict versioning of documents with track changes.

Anonymous input was not used in building the consensus. Instead a Working Group of experts was established, which created strong motivation and willingness to participate, provided a useful variety of professional backgrounds in different disciplines, and allowed us to make use of effective communication and collaboration skills developed over many years. Work was based on efficient and regular telephone conferences and face-to-face meetings dedicated solely to the development of the standard requirements. During the process different opinions were expressed by the experts and minority responses dealt with, but there were no severe disagreeements or diversities of opinion on priorities within the group. Nevertheless, there may be limitations in our approach, which could have been overcome by a more formal consensus technique.

Two further aspects have to be discussed. The first aspect is related to the lack of empirical evidence available to the group in coming to a consensus about the most suitable standard to be stipulated. This particular standard is not alone in this - the other guidelines consulted were also all essentially statements of principle, based on ethical principles and domain expertise. The reason for this is simply that there is no (non-anecdotal) evidence - at least that the working party members were aware of - that relates 'good' or 'bad' practice within trial unit data management and IT to particular policies or systems. This underlines the need for a concrete operational set of standards, such as the ECRIN requirements, because before empirical research can be carried out - measuring some index of overall utility, efficiency, safety (etc.) against the practices employed by units - we have to agree on the important aspects of practice to assess. Most of the existing guidelines and policies are too vague to support such a process. The ECRIN standard, however, is specific enough to potentially be useful for this purpose.

The ECRIN standard should therefore be seen as part of a continuously evolving set of tools, to be employed not just in assessing or accrediting trials units, but able to contribute to future data collection, and thus better identification and understanding of the key aspects of organising systems within trials units.

The fact that the requirements developed, whatever their ultimate source, were filtered through the opinions and expertise of group members, is related to the second aspect - the size of the working group. Though the 25 members of the group could claim considerable experience and expertise between them, they obviously did not cover all types of trials units and all the possible variations in practice.

The working group was very aware of this and was therefore keen to disseminate the standard widely - the principal motivation for submitting this paper. It is envisaged that once available publicly the standard will be the subject of further discussion and will evolve further within a wider framework, In particular the group is anxious to engage national regulatory authorities, with a view to exploring how the standard could be used as widely as possible and embedded in normal practice, rather than being seen as an additional set of requirements only linked to ECRIN activities.

### Certification

The assembled standard requirements list will be the basis for the planned certification of ECRIN data centres, with a certified centre expected to meet all of the minimal requirements. Best practice standards are not mandatory but have been included to provide guidance to data centres who want to improve their data management practices. Certification as an ECRIN approved data centre will demonstrate, first, compliance of the certified centre with regulations and standards, including GCP, second compliance with recommendations of ECRIN in terms of data management, third, that the centre is staffed by expert personnel, and fourth, that the centre is competent in the management of data for international, multicentre clinical trials. Thus, by implementing the certification procedure based on requirements that are GCP-compliant, ECRIN will guaranty a standard quality level for data management performed by academic trial units in pan European trials.

## Conclusions

The standard provided is intended to be an open and widely used set of requirements for GCP-compliant data management and is mainly applicable to academic trial centres. It is intended to be used for quality management, validation, preparation of audits, and for training purposes. The standard can be used by all parties interested in running a data management centre in compliance with GCP and in accordance with European regulations and guidelines. ECRIN will use the requirements as a basis for selection and certification of its ECRIN data centres.

## Competing interests

The authors declare that they have no competing interests.

## Authors' contributions

All authors (members of the ECRIN Working Group on Data Centres) participated in the discussion, evaluation, tuning and harmonisation of the requirements of the standard. CO, WK and SC collected, formulated the requirements and aligned the IT and DM part of the standard. The other members of the ECRIN Working Group on Data Centres were involved in the discussion of the requirements. All authors read and approved the final manuscript.

## Supplementary Material

Additional file 1 **Standard requirements for GCP compliant data management in multinational clinical trials**. Version 1 from 27 May 2010 developed by the European Clinical Research Infrastructures Network (ECRIN) Working Group on Data Centres. Click here for file
