# Supplementary material for: Standard requirements for GCP-compliant data management in multinational clinical trials
Source: Trials. 2011 Mar 22;12:85. doi: 10.1186/1745-6215-12-85 (PMC3074516; doi:10.1186/1745-6215-12-85)
Supplement: Additional file 1 — Standard requirements for GCP compliant data management in multinational clinical trials. Version 1 from 27 May 2010 developed by the European Clinical Research Infrastructures Network (ECRIN) Working Group on Data Centres. [file 1745-6215-12-85-S1.PDF]

EUROPEAN CLINICAL RESEARCH INFRASTRUCTURES NETWORK AND BIOTHERAPY FACILITIES: PREPARATION PHASE FOR THE INFRASTRUCTURE

ECRIN-PPI

FP7 Capacities – Research Infrastructures
ECRIN-PPI No. 211738

Additional file 1

Standard requirements for GCP-compliant data management in multinational clinical trials

Version 1 from 27 May 2010 developed by the European Clinical Research Infrastructures Network (ECRIN) Working Group on Data Centres.

Contents

Introduction 3
Abbreviations 4
IT01. Procurement and Installation (Servers) 6
IT02. Physical Security and Management 6
IT03. Logical Security and Management 7
IT04. Logical Access Control....8
IT05. Business Continuity....9
IT06. General System Validation....9
IT07. Local Software Development....10
IT08. Clinical DBMS Systems....11
IT09. Treatment Allocation Systems....12
IT10. Reporting 12
IT11. Data Export....13
IT12. Importing & Uploading Data 14
IT13. Directly Amending Data....14
IT14. Delivery of Data for Analysis 15
IT15. Long Term (electronic) Data Curation 15
DM01. Clinical Data Management Application - Design and Development....17
DM02. Clinical Data Management Application - Validation 18
DM03. Clinical Data Management Application - Change management 18
DM04. Treatment Allocation and (Un)Blinding Management 19
DM05. Site Management, Training & Support 19
DM06. Data Entry and Processing 20
DM07. Data Quality Checks 21
DM08. Query Management 22
DM09. Data Coding and Standards....22
DM10. Safety Data Management Application 23
DM11. Pre-Analysis Data Management....23
DM12. Managing (physical) Archives 23
IN01. International Aspects 24
ST01. Trials Unit staff competence....24

Introduction

The Working Group on Data Centres of the European Clinical Research Infrastructures Network (ECRIN) has developed a standard describing the requirements of GCP-compliant data management in multinational clinical trials. The standard requirements are based on international, European and national regulations and guidelines relevant to GCP, data security and IT infrastructure, as well as ECRIN documents developed previously. The requirements were developed by expert consensus of the ECRIN Working group on Data Centres, using a structured and standardised process. The requirements are divided into two main parts: an IT part covering standards for the IT infrastructure and computer systems in general, and a Data Management (DM) part covering requirements for data management applications in clinical trials. The standard developed includes 115 IT-requirements, split into 15 separate sections, 107 DM-requirements (in 12 sections) and 13 other requirements (2 sections). Sections IT01 to IT05 deals with the basic IT infrastructure while IT06 and IT07 cover validation and local software development. IT08 to IT015 concerned the aspects of IT systems that directly support clinical trial management. Sections DM01 to DM03 cover the implementation of a specific clinical data management application, i.e. a specific trial, whilst DM04 to DM12 address the data management of trials across the unit. Section IN01 is dedicated to international aspects and ST01 to the competence of a trials unit's staff. Each individual requirement is characterized by an ID number, a precise description, and categorised as either a 'minimal' requirement or 'best practice'.

Abbreviations

AE	Adverse Event
ASCII	American Standard Code for Information Interchange
BC	Business Continuity
BP	best practice
Cat	category of requirement
CDISC	Clinical Data Interchange Standards Consortium (http://www.cdisc.org/)
CDMS	Clinical Data Management System
Citrix	product of Citrix Systems, Inc (desktop virtualisation)
CONSORT	Consolidated Standards of Reporting Trials (http://www.consort-statement.org/)
CRF	Case Report Form
CSV	Comma-Separated Values
DBA	Database Application
DBMS	Database Management System
DDI	Data Documentation Initiative
DM	Data Management
eCRF	electronic Case Report Form
ECRIN	European Clinical Research Infrastructures Network (www.ecrin.org)
eRDC	electronic Remote Data Capture
FDA	Food and Drug Administration (www.fda.gov)
GAMP	Guide for Validation of Automated Systems
GCP	Good Clinical Practice
HTML	Hypertext Markup Language
iCRF	interim Case Report Form
ID	identification number of ECRIN standard requirements
IN	international requirements
IT	Information Technology
MedDRA	Medical Dictionary for Regulatory Activities (www.meddramsso.com)
Min	minimal requirement
ODM	Operational Data Model

PC	personal computer
pCRF	paper-based Case Report Form
PDF	Portable Document Format
PID	personal identifier
QA	Quality Assurance
R	R-Project for Statistical Computing, statistical software (http://www.r-project.org)
SAE	Serious Adverse Events
SAS	statistical software, product of the SAS Institute Inc.
SC	stuff competence requirements
SLA	Service Level Agreement
SOP	Standard Operating Procedure
SPSS	statistical software, product of IBM Corporation
Stata	Data Analysis and Statistical Software, product of StataCorp LP
UI	user interface
UPS	Uninterruptible Power Supply (apparatus that provides emergency power)
USB	Universal Serial Bus (specification to establish communication between devices)
XML	eXtensible Markup Language
XMLschema	description of a XML document

IT 01: Procurement and Installation (Servers)

ID	*Cat.*	*Requirement*
IT01.01	*min*	*Server Specification:* Servers and similar equipment should be specified and selected according to the specific requirements of the trials unit and the functions being supported
IT01.02	*min*	*Server Builds:* Detailed records of builds must be available, for maintenance and safe rebuilding
IT01.03	*min*	*Warranties and Support:* Sufficient support arrangement should be in place for the expected lifetime of the equipment
IT01.04	*bp*	*Server Procurement:* Purchases should show evidence of appropriate selection between alternative suppliers and / or comply with policies stipulated by the host organisation
IT01.05	*bp*	*Procurement Planning:* There should be a defined retirement / replacement policy for servers, given expected lifetimes

Notes

IT01.N1: This section only applies to servers and other high price or specialist items, e.g. network attached storage. It does not apply to the purchase of PCs and laptops, printers and routers and other more standard equipment.

IT01.N2: It is recognised that hardware procurement is very often constrained by the policies of the host organisation (e.g. a University may insist that all servers are purchased from a single supplier).

IT02. Physical Security and Management

ID	*Cat.*	*Requirement*
IT02.01	*min*	*Locked Server Room:* Servers must be housed within a dedicated locked room with unescorted access limited to specified individuals
IT02.02	*min*	*Secured Power Supply:* The power supply to servers should be secured, e.g. by a UPS unit, to allow an orderly shutdown on power failure
IT02.03	*min*	*Encryption of non physically secure data:* no patient data should be stored on anything other than protected servers (e.g. on laptops, desktops, USB sticks etc.) unless it is encrypted
IT02.04	*min*	*Server Failure - Response:* Alerts on server failure within normal business hours should be sent automatically to relevant personnel
IT02.05	*bp*	*Server Failure - Response 24/7:* Alerts on server failure outside of normal business hours should be sent automatically to relevant personnel
IT02.06	*bp*	*Controlled Environment:* Servers should be housed in a temperature controlled environment
IT02.07	*bp*	*Theft and Malicious Damage:* The server room/building should have an alarm system with the alarm linked to a central response centre

IT02.08	*bp*	*Hazard Control - Fire Alarms:* The server room should be fitted with heat and smoke alarms, monitored 24/7
IT02.09	*bp*	*Hazard Control - Fire Response:* The server room should be fitted with automatic fire response measures (e.g. inert gas)
IT02.10	*bp*	*Hazard Control - Water:* Water ingress (e.g. from external flooding) that could inundate power supply / servers should be very unlikely

Notes
IT02.N1: The 'General Note on Scope and Responsibilities', at the beginning of this document is particularly relevant to this list of standards. Many of the functions listed above may be outside the direct control of the trials unit, and formal documents / agreements should therefore be available to provide evidence that the standards are being met.

IT03. Logical Security and Management

ID	*Cat.*	*Requirement*
IT03.01	*min*	*Security Management System:* Regular reviews of IT security systems, practices and documentation, followed by any necessary planning and actions, should occur as part of an ongoing Security Management System
IT03.02	*min*	*Commitment to Data Protection:* The unit or its parent organisation can demonstrate compliance with and commitment to local data protection legislation, including relevant policies, training and individuals with designated roles (e.g. 'Data protection officer')
IT03.03	*min*	*External Firewalls:* External firewalls should be in place and configured to block inappropriate access
IT03.04	*min*	*Encrypted Transmission:* Clinical data transmitted over the internet to or from the trials unit must be encrypted
IT03.05	*min*	*Server Admin Role:* Servers should be protected by a highly restricted administrator password (i.e. known to essential systems staff only)
IT03.06	*min*	*Admin Password Management:* The administrator password should be changed regularly according to locally agreed policies, and stored securely for emergency use (e.g. off site)
IT03.07	*min*	*Server Maintenance:* Necessary patches and updates should be identified and applied in a timely but safe manner to:...<br>the operating system,<br>anti-malware systems,<br>backup systems and<br>major apps (e.g. Clinical DBMSs, Web servers, Remote Access systems, etc.)
IT03.08	*bp*	*Commitment to Information Security:* The unit or its parent organisation can demonstrate management commitment to information security, including relevant groups, policies, training and individuals with designated roles (e.g. 'IT security officer')

IT03.09	*bp*	*Internal Firewalls:* Internal firewalls should be in place and correctly configured, e.g. blocking access to other departments, students
IT03.10	*bp*	*Security Testing:* Regular security testing should be carried out and is documented
IT03.11	*bp*	*Traffic Monitoring:* Traffic activity should be monitored and hacking attempts identified and investigated

Notes
IT03.N1: Again there may be a need for formal documents / agreements (e.g. SLAs) between the data centre and the organisation (e.g. the host university, a hosting service) that may provide or manage many of these facilities.

IT04. Logical Access Control

ID	*Cat.*	*Requirement*
IT04.01	*min*	*Logical Access Procedures:* SOPs and policies for access control to the network(s) and specific systems should be in place
IT04.02	*min*	*Access Control Management:* Each system requiring access controls should have mechanisms, e.g. using roles, group membership, etc., that can be used to effectively differentiate and manage access
IT04.03	*min*	*Granularity of Access:* Access control mechanisms should be granular enough so that users only see the data they need to see
IT04.04	*min*	*Password management:* Network password management should be enforced on all users, including regular password change and password complexity
IT04.05	*min*	*Remote Access:* Remote access (e.g. via Citrix) should be controlled to the same standards as above, and should not normally include access to the host's network
IT04.06	*min*	*Desktop Lockout:* Desktop logins should post a blank screen or screensaver after a locally determined shut down period, and require password re-activation
IT04.07	*min*	*Control - Clinical Data:* Access rights to Clinical Data Systems should be regularly reviewed, changes to access requested and actioned according to defined procedures, by designated individuals, with records kept of all rights, when granted, why and by whom.
IT04.08	*bp*	*Control - General:* Access rights to the network and general should be regularly reviewed, changes to access requested and actioned according to defined procedures, by designated individuals, with records kept of all rights, when granted , why and by whom

Notes
IT04.N1: This section applies to access control for users, both the initial access to the network (often managed by the host organisation) and the specific access required within applications (normally managed using those applications).

IT05. Business Continuity

ID	*Cat.*	*Requirement*
IT05.01	*min*	*Business Continuity Plan:* A Business Continuity plan should be present, covering likely action in the event of a major loss of function (e.g. fire, long term power failure, full server failure, sudden loss of key staff)
IT05.02	*min*	*Back Up Policies:* Documents detailing backup policy, procedures, restores and testing must be in place
IT05.03	*min*	*Back Up Frequency:* Back ups must be taken at least once every 24 hours, using a managed, documented regime
IT05.04	*min*	*Back Up Storage:* Back up media should be stored in a fire proof safe
IT05.05	*min*	*Recovery Testing:* Testing of full restore procedures, back to the original server, should take place at least annually
IT05.06	*min*	*Off site archiving:* The back up regime should involve regular offsite storage of archive media (e.g. monthly)
IT05.07	*bp*	*Business Continuity Integration:* The unit's BC should be integrated with the host organisation's BC plan and appropriate access arranged
IT05.08	*bp*	*Specified Downtime:* A trials unit should state, and adhere to, a specific maximum downtime to any potential user
IT05.09	*bp*	*Business Continuity Review:* Regular review, should occur, at least annually, of the detailed BC plan
IT05.10	*bp*	*Back up - Transaction Logs:* Transaction log backups should take place regularly through the working day, according to a locally agreed plan
IT05.11	*bp*	*Back up - Environment:* The server / DBA environment (groups, log-ins, jobs etc.) should be captured and restorable
IT05.12	*bp*	*Back up - Warm / Hot Failover:* Log shipping or a mirroring procedure should be in place to a warm / hot failover system
IT05.13	*bp*	*Failover testing Recovery:* If available, testing of full restore procedures from a warm / hot failover system should take place at least annually

IT06. General IT System Validation

ID	*Cat.*	*Requirement*
IT06.01	*min*	*Validation Policies:* Policies and SOPs should be in place covering system validation systems and processes
IT06.02	*min*	*Validation master plan:* The unit should have a validation master plan in place, identifying systems, the risks associated with each, and the consequent validation strategy for each

IT06.03	*min*	*Risk based approach:* The general approach to validation of any system should be based on analysis of potential risk, and take into account the system's usage, users and origins
IT06.04	*min*	*Individual validation plans:* Detailed validation plans should exist for any particular system, in line with the master plan and policies described above, detailing the validation required, how and when it should be done, and how it should be recorded
IT06.05	*min*	*Summaries and Recording:* A signed and dated summary of the results of each major validation episode should exist, for each system being validated
IT06.06	*min*	*Detailed Evidence:* More detailed evidence - e.g. of test results or signed user statements - should be available as evidence for the summary validation documents
IT06.07	*min*	*Change Control Policies:* Policies and SOPs should be in place defining change control mechanisms and their scope, who should authorise and review requests, and how they should be documented.
IT06.08	*min*	*Change and Re-validation:* Changes in systems should result in a review of the need for revalidation
IT06.09	*min*	*Software Development:* Evidence should be available that QA processes during software development have been implemented properly

Notes

IT06.N1: These standards apply to general validation of systems, and **not** the specific validation of systems as applied to / used by particular trials. That validation is the subject of DM 02: **Clinical Data Management Application** - Validation and DM 03: **Clinical Data Management Application** – Change management.

IT06.N2: There is no attempt to distinguish validation on different types of systems - e.g. hardware versus software, commercial versus locally developed. The standards above represent an attempt to specify the *general principles* that should be followed and demonstrated by an ECRIN data centre. In practice, different systems will have very different validation requirements - the centre needs to be able to justify the decisions taken.

IT06.N3: A variety of guides and plans exist to assist with validation. The GAMP® "Guide for Validation of Automated Systems", for instance, is one that many people find useful.

IT07. Local Software Development

ID	*Cat.*	*Requirement*
IT07.01	*min*	*Documentation of in-house software:* All modules should be fully documented and specify inputs, outputs, purpose as well as a description of internal mechanisms and algorithms
IT07.02	*bp*	*Code Review:* Regular review and walk through of program code should occur

IT07.03	*bp*	*Re-usable Modules:* A library of reusable validated code / modules / components should be developed
IT07.04	*bp*	*Development Model:* A V-model based procedure is recommended, with constituent modules first validated individually and then integrated before re-validation at the system level
IT07.05	*bp*	*In line Commenting:* All code should have sufficient in line documentation to support tracing of program execution

Notes
IT07.N1: Aside from the initial requirement for comprehensive documentation these standards are really a set of recommendations about software development. The key remains the validation of the software, as covered in IT06. Thus the requirement to validate will be the same, and the requirement to assure properly controlled software development will be the same, whether the software is developed locally or purchased from a commercial software house.

IT08. Clinical DBMS Systems

ID	*Cat.*	*Requirement*
IT08.01	*min*	*Development and Production Instances:* The system offers two instances: development and production
IT08.02	*min*	*Timestamp Control:* Time synchronization within the CDMS is ensured. Sites using eRDC are not able to change the system's time stamp
IT08.03	*bp*	*Metadata Audit Trail:* An audit trail for metadata changes is implemented
IT08.04	*bp*	*Available audit trail:* The audit trail for any particular data item is visible and readable from the user interface for authorised users
IT08.05	*bp*	*Searchable audit trail:* The audit trail is searchable and capable of producing audit trail reports
IT08.06	*bp*	*Development, Production and Test Instances:* The system offers three instances: development, test, production. The test environment and the production environment are identical
IT08.07	*bp*	*Latin Characters:* Systems support a full range of accented Latin characters
IT08.08	*bp*	*Date/numerical Representation:* It is possible to set and use different date and numerical representations in the system

Notes
IT08.N1: These standards refer to the IT specific aspects of the database systems used for clinical trial data, the Clinical DBMSs (or CDMSs).

IT09. Treatment Allocation Systems

ID	*Cat.*	*Requirement*
IT09.01	*min*	*Documentation & Validation:* The underlying logic and operations of all systems for allocating subjects to treatments must be clearly documented and validated
IT09.02	*min*	*Record of Allocation:* A record of all allocation material generated (e.g. randomisation lists) and all decisions made (e.g. within a dynamic balancing system) must be maintained
IT09.03	*min*	*Failover to Manual:* System(s) must be in place, supported by training, to deal with a loss of normal electronic randomisation
IT09.04	*bp*	*Monitoring:* The randomness of list generation or minimisation should be monitored in the context of any particular trial

Notes
IT09.N1: These standards should be read in conjunction with DM04. Treatment Allocation and (Un)Blinding Management

IT09.N2: Documentation of algorithms for treatment allocation should make appropriate reference to the statistical literature. Similarly validation plans should include where possible formal tests of randomness and fitness for purpose drawn from the literature.

IT10. Reporting

ID	*Cat.*	*Requirement*
IT10.01	*min*	*Report access control:* Access to different reports should be controlled and match the users' requirements
IT10.02	*min*	*Report Validation:* The structure and accuracy of reports should be validated against the source data, frequency of validation being determined by a change control process
IT10.03	*min*	*Single Subject Data:* It should be possible to examine and export a full record of a single subject's data (excluding personal identifying data)
IT10.04	*bp*	*Standard Reports:* A set of frequently required (parameterised) reports should be available to appropriate users
IT10.05	*bp*	*UI Ad Hoc Reports:* It should be possible to extract ad-hoc filtered datasets (reports) via the UI
IT10.06	*bp*	*Audit Data:* Selected reports should include the option of including audit related data
IT10.07	*bp*	*Report Rerun:* Once a report is parameterised by user it should be possible to save and rerun it
IT10.08	*bp*	*Metadata included:* The option should exist to include a metadata description of extracted data

IT10.09	*bp*	*Study definition:* Standard reports should include the details of the current study definition in an approved XML schema (trial schedule and data items)
IT10.10	*bp*	*Format of Reports:* Report data can be generated / exported in formats agreed with local report consumers , e.g. PDF, HTML, XML
IT10.11	*bp*	*Data Personnel:* It should be possible to examine and export a record of a single data entry clerk's input data
IT10.12	*bp*	*Key Field Changes:* It should be possible to examine and export a full list of changes to identified key fields, e.g. fields reporting toxicity as part of monitoring
IT10.13	*bp*	*Automatic Generation:* The generation of reports can be automated and can be scheduled

IT11. Data Export

ID	*Cat.*	*Requirement*
IT11.01	*min*	*Data Export Procedures:* SOPs and policies for data exports should be in place
IT11.02	*min*	*Encryption of PID:* The inclusion of any patient identifiable data means any exported file(s) must be encrypted
IT11.03	*min*	*Purpose Recorded:* The purpose of the planned data transfer(s) and the nature of any further processing / transfer planned for the data should be known and logged
IT11.04	*min*	*Assuring Security:* The unit sending the data must have a written agreement / declaration from the recipient that the receiving organisation will maintain appropriate security of data
IT11.05	*min*	*Records of Transfers:* Details of any specific data transfer should be logged, including list of data items, sender, recipient and transfer method, and the date sent
IT11.06	*min*	*Retention of Copies:* Copies of the data sent should be retained within a read only regime and be available as a reference data set for audit / reconstruction purposes
IT11.07	*bp*	*Format of Transfers:* The format of data should be as specified by the recipient
IT11.08	*bp*	*Electronic Archiving*: Standardised formats for electronic archiving (e.g. ASCII, PDF, XML, CDISC ODM, FDA approved SAS format) are used

Notes

IT10.N1: These statements cover the export of any data from the trials unit to a collaborating organisation, e.g. in the context of meta-analysis, or for analysis by an external consultant, when electronic files are used. They are not restricted to electronic transfer, however, and include situations when a USB memory stick is sent by post.

IT12. Importing & Uploading Data

ID	Cat.	Requirement
IT12.01	min	*Upload Procedures:* SOPs and policies for importing / uploading data should be in place
IT12.02	min	*File Retention I:* The original files received should be retained within a read only regime, and be available as a reference data set for audit / reconstruction purposes
IT12.03	min	*Logging of Uploads:* Each upload process should be documented and logged
IT12.04	bp	*File Retention II:* Any files prepared from the originals and used as the direct source of the upload should be kept securely within a read only regime for audit / reconstruction purposes
IT12305	bp	*Data Validation on Input:* Data uploaded to clinical data systems should be checked and annotated as per normal data entry

Notes
IT11.N1: These standards cover uploading bulk data, e.g. from laboratories and collaborators, whether manually, machine or system generated.

IT13. Directly Amending Data

ID	Cat.	Requirement
IT13.01	min	*Requests for Amendment:* Any requests must be in writing and retained, and must include the justification for the change
IT13.02	min	*Recording Amendments:* Any changes made must be logged and the details noted

Notes
IT12.N1: These two standards cover the relatively unusual situation of a data centre being asked to directly amend data in a database, i.e. without using the normal system UI and therefore without the normal audit trail. This may occur if data originally collected outside the system (e.g. from collaborators, monitors, the randomisation system) needs later amendment for some reason. Strict controls must be applied and the related standard statements are given above.

IT14. Delivery of Data for Analysis

ID	*Cat.*	*Requirement*
IT14.01	*min*	*Preparation for Analysis Procedures:* SOPs and policies for generating and preserving datasets for analysis should be in place
IT14.02	*min*	*R/O Analysis Data Retention:* The base data provided for analysis is retained within a read only regime, and is available as a reference data set for any future re-analysis or audit
IT14.03	*min*	*Extracted Data Validation:* The data generated for analysis, and / or the extraction process, should be validated against the source data in the clinical database (not necessarily by IT staff)
IT14.05	*bp*	*Extracted Data - Formats:* The data generated can be generated in Stata, SAS, R and SPSS native formats (as well as CSV, XML)

Notes

IT13.N1: These standards should be read in conjunction with DM11: Pre-Analysis Data Management.

IT13.N2: The notion of locking or freezing a database at the very end of data input is of limited relevance in many trials, where a succession of safety and efficacy analyses are run at different time points, and where data collection may be planned indefinitely (or at least until all subjects have died). The title of this list reflects this.

IT15. Long Term (electronic) Data Curation

ID	*Cat.*	*Requirement*
IT15.01	*min*	*Data Preparation Policies:* Policies / SOPs about *what* data would normally be curated (should normally include metadata, the protocol and other documents as well as all clinical data) should be in place
IT15.02	*min*	*Data Retrieval from Curation:* Policies / SOPs about *how* data would normally be retrieved/ accessed, and who is authorised to do so by the sponsor / investigator, should be in place
IT15.03	*min*	*Data Destruction:* Final destruction of data, if required /allowed, should be as specified by regulations, funding body and/or sponsor
IT15.04	*min*	*Recovery Testing:* The recovery process(es) should be documented and tested
IT15.05	*bp*	*Data Preparation formats:* Data from databases should be decrypted if necessary and transformed into pre-approved XML schemas (e.g. CDISC ODM, Data Documentation Initiative (DDI) 3), or into plain ASCII text files
IT15.06	*bp*	*Data Preparation - Identifiers:* Subject identifiers should be reduced to a minimum or removed altogether, depending on policies / requirements

IT15.07	*bp*	*Data Preparation - Records:* The data preparation process, its inputs, dates and details, should be logged
IT15.08	*bp*	*Additional Material Generation:* Additional electronically stored material may be generated to ensure copies of paper only documents are available (i.e. by scanning)
IT15.09	*bp*	*Curation Facilities:* Service level agreements should be in place with specialist curation providers, providing physical and logically secure long term storage

Notes
IT14.N1: Long term curation generally refers to the permanent storage of data which is no longer being used, but for which restoration and re-access may nevertheless be required in the future. It may involve the removal of the data from the original system. These standards only refer to the processes involved in the *logical* preparation of data for long term curation. The physical and technical aspects of this form of storage are specialised and it is envisaged that centralised and / or third party services would normally be used (as reflected in the relevant statements below).

IT14.N2: This list should be read in conjunction with DM12: Managing (physical) Archives.

DM 01: Clinical data management application - Design and Development

No.	Cat.	Requirement
DM01.01	*min*	*Development Lifecycle Policy*: SOPs covering the development lifecycle of the **clinical data management application** and the CRF (incl. development, testing and deployment) should be in place
DM01.02	*min*	*Design of CRFs*: Process of CRF design is documented, reviewed and includes version management
DM01.03	*min*	*Cross-disciplinary Team*: **Clinical data management application** and CRF development is performed by a cross- disciplinary team (e.g. programmer, trial manager, statistician, data manager)
DM01.04	*min*	*Requirement Specifications of CRF*: The requirements specification for the CRF is driven by the protocol (e.g. primary safety and efficacy variables) and takes into consideration the workflow of trial procedures and organizational aspects
DM01.05	*min*	*Standardized Questionnaires/Instruments*: Validated questions, scales or standard instruments are used where possible (e.g. quality of life questionnaires) and the integrity of validated questionnaires is maintained
DM01.06	*min*	*Data Non-redundancy*: CRF does not duplicate data (e.g. no redundant questions, if not for validation / data management purposes) or calculates results unnecessarily
DM01.07	*min*	*Functional Specifications of CRFs*: CRF functional specifications exist identifying each data item on each CRF (including field names, types, units, validation logic, conditional branching)
DM01.08	*min*	*Checking of* **clinical data management application**: Procedures are implemented for checking (e.g. proofreading) the **clinical data management application** including eCRF and pCRFs against specifications and protocol
DM01.09	*min*	*Delivery of CRFs*: CRFs are delivered to sites prior to enrolment
DM01.10	*min*	*Evaluation of CRF Usability*: The usability of eCRFs is evaluated and assessed before deployment to live environment
DM01.11	*bp*	*Review of CRFs*: CRFs are reviewed against the protocol, end-user expectations and CRF design best practice (e.g. use of validated questionnaires). An acceptance test for CRFs is conducted
DM01.12	*bp*	*Use of Interim CRF*: In cases of eCRF an interim CRF (iCRF) should be available to allow data to be accurately recorded / collated at sites prior to data entry for emergency cases (e.g. if eCRF not available)
DM01.13	*bp*	*Documentation Principles*: Common documentation principles are applied to data items (e.g. preferred coding system, numbering of items, types of missing data, complete answer categories, preference for positive formulated questions, etc.)

DM01.14	*bp*	*Libraries and Metadata Repositories*: Libraries with procedures concerning library management and/or a metadata repository are used, enabling reuse of predefined data items/forms
DM01.15	*bp*	*Quality Management*: Quality documents covering good design practice, usability, local design conventions, etc. are available
DM01.16	*bp*	*User Friendliness of CRFs*: CRFs are divided into appropriate sections with simple and clear instructions for completion and use consistent design principles

DM 02: Clinical data management application - Validation

No.	*Cat.*	*Requirement*
DM02.01	*min*	**Clinical Data Management Application** *Policies*: SOPs and policies for **clinical data management application** and CDMS validation are in place
DM02.02	*min*	*Trial-specific Test Plan*: A trial-specific test plan defines the test methodology, covering scope of test, item pass/fail criteria, etc.
DM02.03	*min*	*Test against Functional Specifications*: The testing with sample data against functional specifications is carried out before deployment to live environment
DM02.04	*min*	*Test of Data Checks*: tests of all validation checks and conditional data capture mechanisms, plus any derivations are conducted, documented and retained
DM02.05	*min*	*Validation Report*: data validation final report for the trial has to be provided and signed by responsible DM person
DM02.06	*min*	*CRF Approval*: approval of the CRF is signed off by key persons
DM02.07	*min*	*Check of Validation Programs, Lists and Scripts*: validation programs, lists and scripts are checked, tested, documented and retained
DM02.08	*bp*	*Validation against Specifications:* the process of **clinical data management application** design and data checks programming is validated against specifications
DM02.09	*bp*	*Validation Report Generation*: system is able to generate reports used for validation

DM 03: Clinical data management application - Change management

No.	*Cat.*	*Requirement*
DM03.01	*min*	*Change Management of* **Clinical Data Management Application**: SOPs and policies for **clinical data management application** change management are in place, including last minute chances
DM03.02	*min*	*Change Management of Metadata*: individual requests for change to metadata (e.g. meta-data, specification of CRF) are justified, itemised and recorded by authorised personnel

DM03.03	*min*	*Amendment for Change*: a risk analysis is conducted before major amendment for change. For each major change the changes, implications and consequent further actions are recorded
DM03.04	*min*	*Test of Amendments*: any amendment is tested in the test environment, following test specifications and the test results are recorded
DM03.05	*min*	*Renewed Training*: in the case of significant changes, the need for re-training is evaluated and implemented if necessary
DM03.06	*min*	*Information of Changes:* mechanisms are implemented to easily inform relevant staff and users of changes, and provide support and explanatory material as required
DM03.07	*bp*	*Requirements for amended CRF*: an amended CRF (that may require ethical approval) has to conform to requested amendments and/or revised protocol. Trial amendments, that may have consequences on the CRF, are taken into consideration
DM03.08	*bp*	*CRF-versioning*: CRF page numbering and version information is always updated to reflect the current status
DM03.09	*bp*	*Management of Change Requests:* change requests are accumulated to minimize amendments

DM 04: Treatment Allocation and (Un)Blinding Management

No.	*Cat.*	*Requirement*
DM04.01	*min*	*Policies for the Implementation of Randomisation*: SOPs and policies for the set up of randomisation in any particular trial are in place
DM04.02	*min*	*Policies for ensuring Randomisation/Blinding*: SOPs and policies exist for protection of blinding and conservation of random allocation to treatment groups
DM04.03	*min*	*Policies for Unblinding*: SOPs are in place to support rapid and safe unblinding of blinded treatments
DM04.04	*min*	*Specification of Randomisation*: specification for the underlying system(s) or the specific trial randomisation process is available
DM04.05	*min*	*Randomisation Implementation*: the randomisation implementation for any particular trial conforms to the protocol
DM04.06	*min*	*Specification of the Randomisation Design*: the study statistician is responsible for the specification of the randomisation design. A randomisation specification document is provided
DM04.07	*min*	*Problem Management of Randomisation*: any problems that arise in the randomisation process are logged and the subsequent actions recorded
DM04.08	*min*	*Randomisation Training*: all staff who handles randomisation requests is adequately trained for each specific trial randomisation process

DM 05: Site Management, Training & Support

No.	*Cat.*	*Requirement*
DM05.01	*min*	*Policies for Site Opening*: SOPs or policies for opening a centre for data collection are in place
DM05.02	*min*	*User Training for Data Entry*: user training with data entry instructions or guidelines, for both pCRFs and eCRFs, is provided for relevant site staff and is documented
DM05.03	*min*	*Test or Productive Environment*: it is clearly indicated to the user whether they are working on a test eCRF or whether the "real trial" has been opened
DM05.04	*min*	*Access to Production System*: site has access to production data systems only once all relevant paperwork and training has been completed; including ethical and research approvals, contracts, site initiation
DM05.05	*min*	*Site Documentation*: after significant changes site documentation is up-dated
DM05.06	*min*	*Responsibility list*: an up to date list of who can do what at each site, including complete CRFs, i.e. a 'delegate log', is maintained

DM 06: Data Entry and Processing

No.	*Cat.*	*Requirement*
DM06.01	*min*	*Data Entry Policies*: SOPs and policies for data entry and corrections are in place
DM06.02	*min*	*Restriction of Data Access*: site staff have access only to data of their site
DM06.03	*min*	*Data Security*: data manager and IT-staff involved will keep data secure and confidential at all times
DM06.04	*min*	*System Security*: system security and access control is ensured, data is only accessible to authorised personnel
DM06.05	*min*	*Tracking of CRFs*: a CRF tracking system is in place.
DM06.06	*min*	*Management of missing CRFs*: systems identify and report on missing or late CRFs /data
DM06.07	*min*	*Quality of Received Data*: data received is checked (pCRF and eCRF)
DM06.08	*min*	*Data Confidentiality*: the blinding of information submitted to the data centre with regard to subject identifying information conforms to national requirements (pseudonymisation)
DM06.09	*min*	*Self Evident Corrections:* clear guidelines and procedures exist to carry out self evident corrections
DM06.10	*min*	*Simple Checks*: simple checks (e.g. range checks) should be available with the possibility to unset for pCRF entry
DM06.11	*min*	*Complex Checks*: complex checks with critical variables (e.g. cross-form validation) are available

DM06.12	*min*	*Audit Trail*: all transactions to the trial database (insert, update, delete) have a clear and complete audit trail, covering the date and time of the input, the person making the change and the old and new values
DM06.13	*bp*	*Timelines for Data Entry*: time-lines for data entry are considered
DM06.14	*bp*	*Amendment/Truncation of Schedules*: logging systems can easily truncate and / or amend schedules to maintain accuracy in identifying outstanding data
DM06.15	*bp*	*Data Deletion*: complete deletion of data from the system is prevented unless it is to comply with a legal request. If indicated for legal reasons, total deletion only takes place using specified procedures and recording with explanatory information

DM 07: Data Quality Checks

No.	*Cat.*	*Requirement*
DM07.01	*min*	*Data Quality Policies*: SOPs and policies are in place regarding data checking, and refer as necessary to the protocol, agreed instructions, GCP and regulatory requirements
DM07.02	*min*	*Batch Validation Checks*: validation checks are able to be executed via a batch process, to identify new warnings, missing, illogical and inconsistent data
DM07.03	*min*	*Data Review*: systems are able to support data checks by generating specified data in formats that match input format (e.g. that mimic CRFs) for manual review of data, e.g. medical consistency checks, lab data pointing to an AE
DM07.04	*min*	*Risk Based Source Data Verification*: a risk based source data verification regime is implemented as specified in the protocol, with the emphasis on primary target variables and other essential data. A check of primary endpoints and other essential data is conducted
DM07.05	*min*	*Documentation of Checks*: All data checking exercises are documented
DM07.06	*min*	*Problem Management*: problems and issues are reported to the appropriate person for query generation or other resolution
DM07.07	*bp*	*Quality Monitoring of Sites*: centres are monitored for quantity / types of errors to identify potential problems, e.g. with particular preset trigger levels
DM07.08	*bp*	*Statistical Evaluation of Data Quality*: statistical methods are used to assess and evaluate data quality (e.g. measures to analyse possible problems and irregularities should cover e.g. multivariate analysis of possible outlier candidates, conspicuous data patterns, preferred numerical sequences, accumulation of values close to defined limits) and the impact on analysis should be evaluated

DM 08: Query Management

No.	*Cat.*	*Requirement*
DM08.01	*min*	*Query Policies:* SOPs and policies are available covering query format, generation, timelines, data change and resolution
DM08.02	*min*	*Query Resolution:* Procedure for resolving of queries exist
DM08.03	*min*	*Query Creation and Tracking*: Queries are created in accordance with specifications and documented procedures. Procedures for tracking of queries exist
DM08.04	*min*	*Responses to Queries*: responses are recorded when returned, identified when outstanding and resent as necessary
DM08.05	*min*	*Actions in Response to Queries*: query resolution tracked and appropriate action taken within agreed timelines and documented in the audit trail
DM08.06	*bp*	*Issuing of Queries*: queries are issued to sites within agreed timelines
DM08.07	*bp*	*Avoidance of Query Duplications*: systems avoids accidental duplication of queries
DM08.08	*bp*	*Generation of Messages*: system is able to generate messages to users not linked to specific data items (i.e. information giving, not expecting a reply)
DM08.09	*bp*	*Generation of Query Reports*: reports are generated showing query generation data, return times etc. broken down by site, by source form, etc.

DM 09: Data Coding and Standards

No.	*Cat.*	*Requirement*
DM09.01	*min*	*Policies for Coding*: SOPs and policies for coding are in place (e.g. to promote consistency and proper use of versions)
DM09.02	*min*	*Coding Training*: coding or categorisation is carried out by personnel trained on the relevant systems
DM09.03	*min*	*Support of CONSORT*: the protocol, **clinical data management application** and CRF, should support the CONSORT trial reporting requirements
DM09.04	*min*	*Coding of SAEs*: The constituent symptoms of all Serious AEs are coded prior to analysis (e.g. MedDRA for drugs)
DM09.05	*bp*	*Use of Standards for Coding*: coding uses named standard systems for particular types of data (e.g. MedDRA) where possible
DM09.06	*bp*	*Consistency of Coding*: coding uses consistent systems across different trials and follow consistent conventions and rules in their use
DM09.07	*bp*	*Coding of AEs*: the constituent symptoms of all AEs should be coded prior to analysis
DM09.08	*bp*	*Autocoding*: use of autoencoder(s) and synonym list(s) where possible, however within well defined limits and with authorisation from senior staff, otherwise manual coding is performed

DM 10: Safety Data Management application

No.	*Cat.*	*Requirement*
DM10.01	*min*	*Policies for Safety Data Management*: SOPs and policies for safety data management are in place
DM10.02	*min*	*Safety Data Management*: safety data management application allow the logging of all forms, faxes and correspondence involved, and subsequent information / evaluation requests
DM10.03	*min*	*Expedited Reporting*: safety data management application supports expedited reporting to authorities
DM10.04	*min*	*Routine Reporting*: safety data management application supports routine reporting to all relevant authorities when required (e.g. annual line listings)
DM10.05	*bp*	*Electronic Reporting*: Safety data management application supports reporting via electronic transfer to authorities
DM10.06	*bp*	*Safety Data Reconciliation*: safety data management application supports the reconciliation of SAEs with other safety data

DM 11: Pre-Analysis Data Management

No.	*Cat.*	*Requirement*
DM11.01	*min*	*Policies for Data Base Locking*: SOPs and policies regarding taking a fixed image of the database (snapshot) and, if required,, 'locking' and 'unlocking' databases are in place. In case a locked database is unlocked a documented reason is provided
DM11.02	*min*	*Data Completion*: all relevant data (or all except for a pre-defined / pre-agreed fraction) have been received prior to data extraction for analysis (database lock)
DM11.03	*min*	*Query resolution completion*: All queries (or all except for a pre-defined / pre-agreed fraction) have been resolved
DM11.04	*min*	*Data Reconciliation*: all external data (e.g. safety database, lab data) has been reconciled
DM11.05	*min*	*Data Base Consistency Check*: relevant batch consistency checks of database have been completed and actioned
DM11.06	*bp*	*Review of Coding*: all relevant coding has been reviewed
DM11.07	*bp*	*Data Base Audit*: database audit should be carried out, documenting error rate

DM 12: Managing (physical) Archives

No.	*Cat.*	*Requirement*
DM12.01	*min*	*Policies for Archiving*: SOPs and policies are in place concerning physical archiving of essential trial documents
DM12.02	*min*	*Access to Archive*: access to study archive is documented
DM12.03	*min*	*Protection of Archive*: measures are in place to guarantee safe archiving

		(e.g. locked rooms and fire-proof cupboards, safe area, protected and controlled access for authorized staff only)
DM12.04	*min*	*Archiving Duration*: essential trial documents (including data) are archived for as long as specified by protocol, regulations, funding body and/or sponsor
DM12.05	*min*	*Trial Reconstitution*: conduct of trial can be reconstituted from archived essential trial documents

IN 01: International Aspects

No.	*Cat.*	*Requirement*
IN01.01	*min*	*User Support*: eRDC Help Desk and Hot Line is provided covering user hours
IN01.02	*bp*	*CRF Translation*: if necessary, CRFs/eCRFs can be translated into the language(s) required for the trial, including messages associated with error checking. Translations are verified
IN01.03	*bp*	*Support of National Regulations*: application display, change or hide questions / CRFs to better support national legislation (without using different versions)
IN01.04	*bp*	*Multilingual User Support*: help desk and hot line can deal with the language of the users and provide some sort of help

SC01: Trials Unit staff competence

No.	*Cat.*	*Requirement*
SC01.01	*min*	*Policies for Training*: SOPs and policies are in place describing induction and training requirements / policies / procedures
SC01.02	*min*	*Staff Competence*: DM-staff is competent, trained or being trained to do the job(s) required of them
SC01.03	*min*	*Documentation of Training*: records of training are kept for all DM-staff, kept centrally and / or by the staff themselves
SC01.04	*min*	*Staff Support*: help and support for DM-staff is available
SC01.05	*bp*	*Planning of Staff Training*: training plans are linked to annual appraisal
SC01.06	*bp*	*Ticketing System*: a formal mechanism for requesting support and logging requests / actions should exist
